# Dissecting apoptosis the omics way

**DOI:** 10.7554/eLife.01587

**Published:** 2013-11-12

**Authors:** Petra Van Damme

**Affiliations:** 1 **Petra Van Damme** is at the Department of Medical Protein Research, VIB, and Department of Biochemistry, Ghent University, Ghent, Belgiumpetra.vandamme@vib-ugent.be

**Keywords:** apoptosis, proteomics, ribosome profiling, caspase, myeloma, Human

## Abstract

A combined analysis of transcription, translation and protein degradation reveals the global effects of an anticancer drug on tumour cells.

**Related research article** Wiita AP, Ziv E, Wiita PJ, Urisman A, Julien O, Burlingame AL, Weissman JS, Wells JA. 2013. Global cellular response to chemotherapy-induced apoptosis. *eLife* **2**:e01236. doi: 10.7554/eLife.01236

**Image** Heat map showing the increase in the relative abundance of a small subset of proteins triggered by the chemotherapeutic drug bortezomib

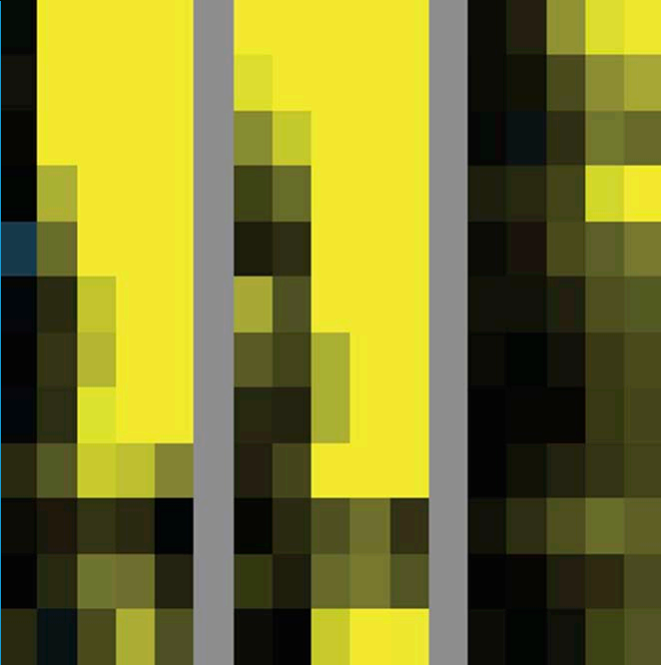

Most anticancer drugs work by triggering tumour cells to undergo programmed cell death, or apoptosis. However, many tumour cells activate a stress response in an attempt to combat the effects of the drugs. To date, most studies that have examined this response have focused on how the drugs affect the production of messenger RNA (mRNA) via transcription in the tumour cells, and much less is known about the effects of these drugs on the translation of the mRNA, or on proteins. Now, in *eLife*, James Wells of the University of California, San Francisco and co-workers—including Arun Wiita as first author—use a new approach to study changes in transcription, translation and protein degradation in tumour cells exposed to an anticancer drug. They show that by targeting key regulators of the stress response, they can lower the threshold for triggering apoptosis in the tumour cells (Wiita et al., 2013).

To study apoptosis in detail, Wiita et al. used a systems-level approach to examine transcription, translation and protein degradation (proteolysis) in a myeloma cell line—a type of cancer of white blood cells—exposed to an anticancer drug called bortezomib. They paired mRNA sequencing with a technique called ribosome profiling, in which the presence of a ribosome bound to mRNA (a ‘footprint’) is used to reveal which mRNAs are being translated. This technique has proven to be indispensable for the genome-wide study of translation (Ingolia et al., 2009). Wiita et al. used an additional suite of tools to study the abundance of proteins and the rate at which they were broken down by apoptotic proteases (Mahrus et al., 2008; Agard et al., 2012).

By comparing the number of ribosome footprints with the number of mRNA sequencing reads, it was possible to deduce the efficiency of translation within the tumour cells (Figure 1). In line with previous reports, they found that bortezomib—which inhibits a complex called the proteasome—caused misfolded proteins to accumulate inside cells. As a result, the endoplasmic reticulum (which is involved in protein trafficking) became overwhelmed, triggering the unfolded protein response (Obeng et al., 2006). This leads to a series of cellular processes to counteract the accumulation of misfolded proteins, including increased protein folding, inhibition of translation, and, as a last resort, the induction of apoptosis. Witta et al. showed that bortezomib increased both transcription and translation of a number of genes involved in the folding or degradation of proteins, and downregulated the expression of genes involved in cellular proliferation.

**Figure 1.**
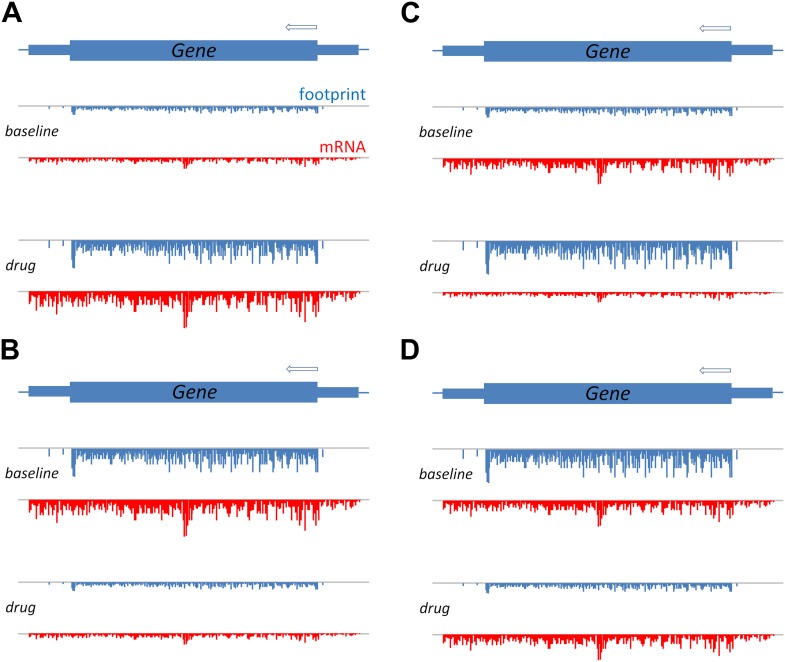
A schematic to show how translation efficiency can be assessed by means of ribosome profiling. The production of a protein from a gene involves transcription, which can be measured in terms of mRNA sequence reads (red), and translation, which can be measured in terms of ribosome footprints (blue). (**A**) Relative to a baseline condition (top), bortezomib increases both the transcription and the translation of certain genes, as can be seen from the mRNA levels and the ribosome footprints (bottom). (**B**) Bortezomib can also decrease both transcription and translation. For other genes, bortezomib had differing effects on these two processes: an increase in protein levels relative to baseline, despite a reduction in mRNA, indicates that the efficiency of translation is increased (**C**); and a reduction in protein levels relative to baseline, despite no change in mRNA, indicates that the efficiency of translation is reduced (**D**).

The coding sequence of genes is preceded by an upstream regulatory region called the 5′-leader. Ribosomes have frequently been shown to initiate translation from within this 5′-leader when cells are exposed to oxidative stress (Gerashchenko et al., 2012) or undergo differentiation (Ingolia et al., 2011), thereby providing another level of translational control. Ribosome occupancy of the 5′-leader sequence may indicate the translation of short regulatory polypeptides encoded by upstream open reading frames, or point to the expression of alternative isoforms of the protein extended at the N-terminus (Ingolia et al., 2011). Wiita et al. found that genes whose expression was regulated by bortezomib showed altered translation from the 5′-leader compared to non-regulated genes. Nevertheless, despite the relatively large changes in mRNA and footprint reads that were seen across the tumour cell genes, the vast majority of proteins showed no changes in their relative abundance, as inferred from quantitative proteomics data.

Based on estimates of the absolute levels of transcripts and proteins, Wiita et al. then devised a model to account for the limited changes in protein levels relative to those of mRNA and mRNA-translation events. Their proposed ‘mass-action model of translation’ describes changes in the number of copies of a protein per cell as a function of the number of copies of a transcript per cell, as well as the rates of mRNA translation and protein degradation. The model demonstrates that increases in the abundance of proteins tend to be coupled to increases in absolute transcript levels, and implies that for the same increase in relative transcript levels, low abundance proteins will undergo a larger increase in relative protein abundance than will high abundance proteins. Finally, the results of Wiita et al. revealed that proteins that were upregulated in response to bortezomib were not preferentially processed by proteases relative to other proteins, suggesting that the changes in transcription and translation do not affect the dynamics of protein processing once apoptosis ensues.

Myeloma cells produce large quantities of antibodies and have therefore adapted to cope with an increased burden of secretory protein synthesis and folding, but these changes have also made them particularly sensitive to the effects of proteasome inhibition. Although cells can adapt to protein folding insults following chronic exposure (Jager et al., 2012), the acute nature of bortezomib treatment is likely to prevent such adaptation, despite the translational re-programming observed in bortezomib treated myeloma cells. Adaptation has also been linked to low levels of activation of the stress response and a distinct pattern of protein expression (Rutkowski et al., 2006). Since adaptation could underlie the ability of cells to become resistant to chemotherapy, it would be interesting to compare the responses of resistant and sensitive cells. It also remains to be explored how the actions of bortezomib relate to those of other chemotherapeutic drugs and other apoptosis inducing agents. Overall, it is likely that efforts to integrate the transcriptome, translatome, proteome and degradome responses will typify future systems biology research and lead to new molecular insights into cancer and the drugs used to treat it.
